# Association of Dietary Inflammatory Index and Thyroid Function in Patients with Hashimoto’s Thyroiditis: An Observational Cross–Sectional Multicenter Study

**DOI:** 10.3390/medicina60091454

**Published:** 2024-09-05

**Authors:** Sanja Klobučar, Gordana Kenđel Jovanović, Jadwiga Kryczyk-Kozioł, Maja Cigrovski Berković, Jelena Vučak Lončar, Nikolina Morić, Katarina Peljhan, Dario Rahelić, Dunja Mudri, Ines Bilić-Ćurčić, Tatjana Bogović Crnčić

**Affiliations:** 1 Department of Endocrinology, Diabetes and Metabolic Diseases, Clinical Hospital Centre Rijeka, 51000 Rijeka, Croatia; sanja.klobucarm@gmail.com; 2 Faculty of Medicine, University of Rijeka, 51000 Rijeka, Croatia; tatjanabc@medri.uniri.hr; 3 Department of Health Ecology, Teaching Institute of Public Health of Primorje—Gorski Kotar County, 51000 Rijeka, Croatia; gogakendel@gmail.com; 4 Department of Food Chemistry and Nutrition, Jagiellonian University Medical College, 31-008 Kraków, Poland; jadwiga.kryczyk@uj.edu.pl; 5 Department for Sport and Exercise Medicine, University of Zagreb, Faculty of Kinesiology, 10000 Zagreb, Croatia; maja.cigrovskiberkovic@gmail.com; 6 Department of Health Studies, University of Zadar, 23000 Zadar, Croatia; jelenaby23@yahoo.com; 7 Department of Endocrinology, Zadar General Hospital, 23000 Zadar, Croatia; 8 Health Center of Primorje—Gorski Kotar County, 51000 Rijeka, Croatia; nikolinamorich@gmail.com; 9 Department of Dermatology, Clinical Hospital Centre Rijeka, 51000 Rijeka, Croatia; katarinapeljhan@gmail.com; 10 Faculty of Medicine, J.J. Strossmayer University Osijek, 31000 Osijek, Croatia; dario.rahelic@gmail.com (D.R.);; 11 Endocrinology and Metabolic Diseases, Vuk Vrhovac University Clinic for Diabetes, Merkur University Hospital, 10000 Zagreb, Croatia; 12 School of Medicine, Catholic University of Croatia, 10000 Zagreb, Croatia; 13 Clinical Institute for Nuclear Medicine and Radiation Protection, Clinical Hospital Center Osijek, 31000 Osijek, Croatia; 14 Department of Endocrinology, Clinical Hospital Center Osijek, 31000 Osijek, Croatia; 15 Clinical Department of Nuclear Medicine, Clinical Hospital Centre Rijeka, 51000 Rijeka, Croatia

**Keywords:** body mass index, diet, dietary inflammatory index, Hashimoto’s thyroiditis, inflammation, thyroid

## Abstract

*Background and Objectives:* The available research suggests that dietary patterns with high inflammatory potential, as indicated by a high DII score, may exacerbate inflammation and potentially influence thyroid function. Therefore, the aim of this study was to investigate the associations between the inflammatory potential of a diet and thyroid function in adults with Hashimoto’s thyroiditis (HT). *Materials and Methods:* A total of 149 adults diagnosed with Hashimoto’s thyroiditis were enrolled in this observational, cross-sectional, multicenter study. The Dietary Inflammatory Index (DII^®^) was calculated using a 141-item food frequency questionnaire (FFQ). The serum levels of the thyroid-stimulating hormone (TSH), free thyroxine (fT4), thyroid peroxidase antibodies (TPO-Ab), and high-sensitivity C-reactive protein (hsCRP) were determined. *Results:* The DII^®^ scores ranged from −3.49 (most anti-inflammatory) to +4.68 (most pro-inflammatory), whereas three DII^®^ tertile ranges were defined as <−1.4, −1.39 to +1.20, and >+1.21, respectively. Participants in tertile 1 (more anti-inflammatory diet) had significantly higher levels of fT4 than those adhering to a more pro-inflammatory diet (*p* = 0.007). The levels of hsCRP and TSH appeared to increase with increasing the DII^®^ score, but without statistical significance. A significant association was found between the DII^®^ and TSH (β = 0.42, *p* < 0.001) and between DII^®^ and free thyroxine (β = 0.19, *p* < 0.001). After adjustment for age, gender, energy intake, and physical activity, a significant positive correlation remained between the DII^®^ and TSH (β = 0.33, *p* = 0.002) and between the DII^®^ and body mass index (BMI) (β = 0.14, *p* = 0.04). *Conclusions:* Adherence to an anti-inflammatory diet appears to be beneficial in patients with Hashimoto’s thyroiditis, suggesting that dietary modification aimed at lowering DII^®^ levels may be a valuable strategy to improve clinical outcomes in these patients.

## 1. Introduction

Hashimoto’s thyroiditis (HT), also known as chronic lymphocytic thyroiditis, is one of the most frequent autoimmune diseases worldwide. Its global prevalence in adults is reported to be 7.5%, with 17.5% in women and 6.0% in men [1]. It primarily affects middle-aged women [2] but can also occur in men and women of any age and in children. In the vast majority of cases, this autoimmune disease has a subtle onset and generally tends to evolve toward hypothyroidism [3]. It is well established that thyroid hormone status correlates with body weight and that patients with thyroid dysfunction may experience changes in body weight and body composition. Thyroid hormones influence key metabolic pathways that control energy balance by regulating energy storage and expenditure. Weight gain or difficulty losing weight is strongly associated with hypothyroidism [4,5]. Even mild thyroid dysfunction in the form of subclinical hypothyroidism is found to be linked to significant changes in body weight [6]. While obesity is generally considered secondary to thyroid dysfunction, several lines of evidence suggest that changes in thyroid-stimulating hormone (TSH) levels may be secondary to obesity. So far, studies have revealed a link between obesity and thyroid autoimmunity, with the adipocyte hormone leptin appearing to be the key factor linking these two conditions [7].

From a clinical perspective, Hashimoto’s thyroiditis and obesity frequently coexist. The link between these two common clinical conditions have become more relevant in the context of a dramatic increase in the prevalence of obesity worldwide [8,9]. Obesity is associated with an increase in various inflammatory markers, both locally in adipose tissue and systemically, resulting in chronic low-grade inflammation. This sustained inflammation is thought to impair several regulatory pathways, leading to the disruption of human self-tolerance mechanisms, a decrease in circulating Tregs, and an increase in Th1 and Th17 cells, creating the environment for the development of autoimmune disorders, including Hashimoto’s thyroiditis [10,11].

Diet quality plays a critical role in modulating inflammation and influencing the risk of chronic diseases and body weight management. The potential pro- or anti-inflammatory properties of dietary patterns and individual dietary components are fundamental to understanding the influence of diet in preventing and managing chronic diseases, highlighting the need for public health strategies that promote nutritious, anti-inflammatory eating patterns. The Dietary Inflammatory Index (DII^®^) is a tool for evaluating the inflammatory potential of a diet and an increasing body of evidence has shown the association between a high DII^®^ score (indicating a pro-inflammatory diet) and chronic disease risk, including cardiovascular disease, type 2 diabetes, metabolic syndrome, and obesity [12,13,14,15]. Targeting the immune system responses through diet modulation may alleviate the inflammatory burden and enhance immune responses. However, limited studies are available on the association of the inflammatory potential of a diet with thyroid function [16,17].

Understanding the relationship between DII and hypothyroidism can inform dietary recommendations and interventions aimed at improving thyroid health and managing symptoms effectively. Therefore, the aim of this study was to investigate the associations between the inflammatory potential of a diet and thyroid function in adults with Hashimoto’s thyroiditis.

## 2. Materials and Methods

### 2.1. Study Participants

A total of 149 adult patients with Hashimoto’s thyroiditis, aged 19 to 72, were included in this observational, non-interventional, cross-sectional, multicenter study. Of these, 140 were female and 9 were male. Ninety-five participants used levothyroxine replacement therapy. To include an eligible number of subjects with HT in this study, recruitment was performed from five Croatian thyroid outpatient clinics during nine months in 2022. The calculation of required number of patients was based on HT global prevalence of the disease in the population of 7.5% [1], with an assumed significance level of 0.05 and the power of the study at 80%. Physicians were asked to enroll eligible patients consecutively in order to avoid selection bias. The exclusion criteria were as follows: subclinical or overt hypothyroidism, chronic heart, kidney, and/or severe liver disease, malignant disease or history of malignant disease, pregnancy, and lactation. Additional exclusion criteria included usage of any immunosuppressive medication; nutritional or other hormone supplements, including liothyronine and oral contraceptives; anti-inflammatory drugs or being on dietary restrictions for at least three months prior to study inclusion; and no history of thyroid surgeries (a potential confounding factor influencing the LT4 requirements necessary to normalize TSH levels).

A self-administered questionnaire was used to collect data on patient demographic and lifestyle characteristics including marital status, education level, occupation level, the usage of anti-inflammatory medications, and smoking status. To minimize bias, each patient was informed of the study background and aims by a medical specialist and instructed how to fill out the questionnaire by an educated dietitian who explained in detail the physical activity questions and dietary habits questions. For assessing physical activity, a validated questionnaire used in the European Prospective Investigation into Cancer and Nutrition (EPIC) was applied [18]. Based on questionnaire results, participants were classified as having low, moderate, or high physical activity levels. Weight measurement was performed using a digital scale (Seca, Hamburg, Germany) with an accuracy of 0.1 kg, and height was measured with a wall-mounted stadiometer with a sensitivity of 0.1 cm (Seca, Hamburg, Germany). Body mass index (BMI) was calculated by weight in kilograms divided by height in meters square. The study was approved by the Ethics Committee of the Clinical Hospital Center Rijeka (approval certificate registry number 003-05/16-1/40) and was conducted in accordance with the Declaration of Helsinki. All the participants gave written informed consent prior to participation and data collection.

### 2.2. Blood Samples and Laboratory Assessments

Whole venous blood samples of study participants were collected in the morning, after 12 h fasting period. Serum levels of thyroid-stimulating hormone (TSH), free thyroxine (fT4), and thyroid peroxidase antibodies (TPO-Ab) were analyzed on Immulite 2000 xp, Siemens Healthcare Diagnostics Limited, Frimley, Camberley, Surrey, UK, while high-sensitivity C-reactive protein (hsCRP) was analyzed on Olympus 5800, Westborough, MA, USA, with standard method using specific commercial kits.

### 2.3. The Assessment of Dietary Intake

Dietary intake was assessed with a validated food frequency questionnaire (FFQ) that included 141 foods and beverages. Participants recorded their food and beverage frequency and average intake for the previous month. Frequency of food consumption was reported in six categories: once a day, 1–2 times a week, 3–4 times a week, 5–6 times a week, once a month, and 2–3 times a month, while the average intake of food and beverages was reported as small, medium, and large.

### 2.4. The Inflammatory Potential of Participants’ Diet

The inflammatory potential of participants’ diets was evaluated by the Dietary Inflammatory Index (DII^®^), which included the following dietary variables: energy, proteins, total fat, saturated fat, monounsaturated fatty acids, polyunsaturated fatty acids, omega 3 fatty acids, omega 6 fatty acids, trans fatty acids, cholesterol, carbohydrates, dietary fiber, alcohol, beta-carotene, vitamin A, vitamin D, vitamin E, vitamin C, vitamin B_1_, vitamin B_2_, vitamin B_3_, vitamin B_6_, vitamin B_9_, vitamin B_12_, calcium, iron, magnesium, zinc, selenium, iodine, caffeine, flavan-3-ol, flavones, flavonols, flavonones, and anthocyanidins [12]. The DII^®^ of the study participant was calculated by subtracting each food parameter from the “standard global mean” and dividing the result by the standard deviation to obtain “z” scores. To reduce the impact of “right skewing”, “z” scores were then translated into percentile scores, doubled, and subtracted by 1′ [12]. These computed values were then multiplied by the inflammatory effect score to obtain the overall DII^®^ score. A lower DII^®^ score than 0 indicated a more anti-inflammatory diet, while a higher DII^®^ score indicated a more pro-inflammatory diet.

### 2.5. Statistical Analysis

Continuous variables are presented as means and standard deviations, while categorical variables are presented as percentages. Differences were estimated using ANOVA, Kruskal–Wallis test, or chi-square tests depending on the type of variable. The multivariable-adjusted model included age, gender, energy intake, and physical activity as potential confounders. All presented *p* values are two-tailed, and differences were considered statistically significant at *p* < 0.05. For all statistical analyses, the data analysis software system Statistica, version 13 (TIBCO Software Inc., Palo Alto, CA, USA, 2017) was used.

## 3. Results

A total of 149 adults diagnosed with Hashimoto’s thyroiditis, with an average age of 47.4 (SD + 12.9) and an average BMI of 28.3 (SD + 5.9) kg/m^2^, were included in this study. There were 81 participants (54.4%) who had a DII^®^ score less than 0, meaning they had a diet with an anti-inflammatory potential. The average DII^®^ score was 0.03 (SD + 2.63), indicating a diet with a pro-inflammatory potential. The DII^®^ scores ranged from −3.49 (most anti-inflammatory) to +4.68 (most pro-inflammatory), whereas the three DII^®^ tertile ranges were <−1.4 (T1), −1.39 to +1.20 (T2), and >+1.21 (T3). Participants’ characteristics by DII^®^ tertiles are shown in Table 1. There were no significant differences across categories of DII^®^ regarding age, gender, marital status, education level, occupation level, smoking status, usage of anti-inflammatory medications, and body mass index. Participants in the second DII^®^ tertile reported lower physical activity levels compared to those in the other tertiles (*p* = 0.008)

Mean values for C-reactive protein and thyroid function markers across categories of DII^®^ are presented in Table 2. Participants in tertile 1 (lowest DII^®^ scores) had significantly higher levels of fT4 compared with participants in tertiles 2 and 3 (*p* = 0.007). Even though TSH, fT4, and hsCRP variations remained within the reference range, the levels of hsCRP and TSH seemed to rise with an increasing DII^®^ score, though this increase was not statistically significant. Thyroid peroxidase antibody levels did not reach statistical significance, but they were more than twice as high in participants with higher DII^®^ scores than in those with lower scores.

The results for energy and nutrient intakes are summarized in Table 3. There was a significant difference in DII^®^ scores across tertiles, with T1 showing the most anti-inflammatory potential and T3 showing the least (*p* < 0.001). A decreasing trend in energy intake was observed from T1 to T3 (*p* < 0.001). Protein intake as a percentage of energy intake increased significantly from T1 to T3 (*p* = 0.02), while saturated fat intake was significantly higher in T2 compared to T1 and T3 (*p* = 0.04). On the other hand, carbohydrate intake decreased significantly (*p* < 0.001), whereas the intake of Omega-3 and Omega-6 fatty acids significantly decreased across tertiles, (*p* < 0.001 and *p* = 0.002, respectively). Significant decreases in the intake of vitamins and minerals, such as vitamins A, D, E, and C; calcium; iron; magnesium; zinc; selenium; and iodine, were observed from T1 to T3 (all *p* < 0.001 except for vitamin D with *p* = 0.01). In addition, significant decreases in flavones, flavonols, flavonones, and anthocyanidins were present across tertiles, indicating a decrease in the intake of anti-inflammatory phytochemicals (*p* < 0.001 for all).

Linear regression analysis revealed a positive association between DII^®^ and TSH (β = 0.42, *p* < 0.001) and a negative one between DII^®^ and free T4 (β = 0.19, *p* < 0.001). After adjustment for age, gender, energy intake, and physical activity, a significant positive relationship remained only between DII^®^ and TSH (β = 0.33, *p* = 0.002), while the association between DII^®^ and free T4 was lost. In addition, a significant positive association was observed between DII^®^ and body mass index (β = 0.14, *p* = 0.04) in the adjusted model, which is shown in Table 4.

## 4. Discussion

This observational, cross-sectional, multicenter study aimed to investigate the relationship between the inflammatory potential of a diet and thyroid function in adults with Hashimoto’s thyroiditis.

Our data revealed that most demographic variables, such as age, BMI, gender, marital status, education, and occupation, did not significantly differ across the Dietary Inflammatory Index tertiles as opposed to physical activity levels. The higher prevalence of low physical activity in the middle tertile suggests a potential link between physical activity and dietary inflammatory potential, which is in line with previous research demonstrating that diets with lower inflammatory potential, indicated by low DII^®^ scores, are often associated with higher levels of physical activity. This relationship implies that individuals engaging in regular physical activity may also adhere to dietary patterns that reduce inflammation, thereby synergistically reducing the risk of inflammation-related conditions, such as cardiovascular diseases and autoimmune disorders [12].

In the present study, adults with HT adhering to a more anti-inflammatory diet appeared to have lower TSH levels, higher free T4 levels, and lower BMI values, suggesting favorable effects of an anti-inflammatory dietary approach on thyroid function and metabolic health. A significant association between the Dietary Inflammatory Index and free T4 suggests that a more pro-inflammatory diet might negatively impact thyroid hormone levels. While CRP and TSH levels did not show significant differences across tertiles, the observed trends warrant further investigation. However, a significant positive correlation between DII^®^ and TSH was observed, which remained after adjustment for age, gender, energy intake, and physical activity, as opposed to the DII^®^ and FT4 correlation, which was lost. These findings are in line with Chen’s study [19]. In another study, only FT3 and TT4 remained positively associated with DII after adjustments for different confounders [20]. In addition, a recently published cross-sectional study investigating an association between dietary inflammatory potential and thyroid function in US adult males revealed that a more pro-inflammatory diet was associated with higher total T4 levels in men [16]. More importantly, in our study, the highest protein intake was observed in participants in the highest tertile of DII^®^, whereas in the US study, participants in the highest tertile of the DII^®^ (more pro-inflammatory diet) had a relatively lower protein intake [16]. This may be explained, at least in part, by gender-related differences in dietary habits, as the majority of participants in our study were women. Women are more often affected by Hashimoto’s thyroiditis, and the female-to-male ratio is 18:1. Moreover, recent research has found that diets rich in animal protein elicit a pro-inflammatory response, suggesting a link between higher protein intake and a higher DII^®^, as confirmed in our study [21]. Also, a T3 tertile group with a most pro-inflammatory diet had the lowest intake of carbohydrates and related energy share, while, at the same time, having the highest intake of proteins and related energy share as well. This group of patients was the youngest, with the highest BMI values, the lowest level of physical activity, and the most unfavorable biomarkers of inflammation and thyroid function. A generally lower food intake and energy intake was observed compared to the other two groups in unfavorable proportions regarding macronutrient energy share. Thus, we could hypothesize that this group of patients, probably on their own initiative, followed a low-energy high-protein dietary pattern, possibly to reduce body weight because they had the highest value and range of BMI value.

The underlying mechanism could be the activation of the innate immune system by dietary patterns that are high in refined carbohydrates, sugars, and saturated and trans fatty acids and low in fiber, antioxidants, and omega-3 fatty acids. This could result in an excess of pro-inflammatory cytokines and a decrease in anti-inflammatory cytokines [22]. In the research conducted by De Rosa et al., it has been proposed that the chronic overactivation of the mTOR pathway leads to impaired proliferation potential of Treg cells [23]. As a result of obesity and nutritional overload, the balance between Treg cells and pro-inflammatory TH1 and TH17 cells is disturbed, leading to loss of metabolic homeostasis and the development of autoimmunity. The chronic low-grade inflammation associated with the above dietary patterns and consequent development of autoimmunity is triggered at the molecular level by the activation of several signaling pathways, which includes signal transducer and activator of transcription 3 (STAT3), IκB kinase (IKK), matrix metallopeptidase 9 (MMP9), mitogen-activated protein kinases (MAPK), cyclooxygenase 2 (COX2), and nuclear factor kappa-light chain enhancer of activated B cells (NF-Kβ) [24].

Another pathophysiological mechanism involves a positive influence of diets with low DII^®^ scores on gut microbiota composition. A balanced gut microbiota is crucial for maintaining systemic immune balance. In particular, it is observed that microbiota dysbiosis stimulates autoimmune processes in patients with HT. Patients with HT are at greater risk of developing intestine bacterial overgrowth because fluctuations in thyroid hormone levels can affect the composition of the gut microbiota. Studies have shown that HT patients also have increased intestinal permeability compared to the control group [25]. Understanding the interplay between DII^®^ scores and gut microbiota could provide insights into dietary interventions that could mitigate inflammatory processes and support thyroid health in individuals with Hashimoto’s thyroiditis [26,27,28].

Dietary factors with a particular impact on thyroid function include elements such as iodine, iron, and selenium, and the best dietary sources, which are meat and meat products, fish, and seafood [29]. HT is associated with the inadequate intake of selenium, iron, magnesium, zinc, iodine, vitamins A, B, C, D, E, and also omega-3 fatty acids [30]. This study found that participants who had a diet with a more anti-inflammatory potential also had significantly higher intakes of iodine, iron, and selenium than those with a more pro-inflammatory diet. Linear regression analysis after adjustment for age, gender, energy intake, and physical activity revealed a significant positive association between iodine and the DII^®^, and also between selenium and the DII^®^, but not iron. Impaired selenium status has been known to cause thyroid hormone synthesis dysfunction [31]. The intake of selenium is necessary to maintain the integrity of the thyroid gland exposed to H_2_O_2_, excessive stimulation by TSH or TSH receptor-stimulating antibodies (TRAb), or immune system attack in cases of autoimmune thyroiditis, as well as to ensure a normal thyroid hormone profile [32].

Furthermore, participants with lower DII^®^ scores had a higher consumption of omega-3 and omega-6 fatty acids than participants with higher DII^®^ scores, which probably contributed to the anti-inflammatory potential of the diet. These results are in line with the emerging research, which suggests that diets with a low DII^®^ score, which include higher omega-3 and balanced omega-6 fatty acid intake, may reduce inflammation and modulate immune responses, potentially ameliorating the symptoms and progression of Hashimoto’s thyroiditis [33]. Possibly the best option would be the Mediterranean diet rich in fruits, vegetables, whole grains, nuts, legumes, fish, and olive oil, while being low in red meat and processed foods [34]. These components are saturated with essential nutrients, antioxidants, and anti-inflammatory agents, which help in maintaining physiological balance. The Mediterranean diet helps decrease allostatic load by lowering systemic inflammation and oxidative stress [35], promotes cardiovascular health by improving lipid profiles and reducing blood pressure, and stabilizes blood sugar levels by preventing insulin resistance. Additionally, the diet’s impact on the gut microbiome through the consumption of fiber-rich foods supports mental well-being [36].

Findings from our study underscore the importance of dietary composition in modulating the inflammatory potential of the diet, as higher DII^®^ scores (indicating more pro-inflammatory diets) were associated with lower intakes of beneficial nutrients and phytochemicals, which may influence the management of inflammatory conditions such as Hashimoto’s thyroiditis.

The present study has some limitations that should be considered when interpreting the results. The nature of the study is cross-sectional; therefore, it cannot be used to infer causality. Subsequent large case–control and/or cohort studies are important to further confirm the results. In addition, participants may have misreported their dietary habits, as responses in the FFQ depend on the participants’ recollection of food intake. Still, recall bias could not be completely avoided, although a trained nutritionist explained the meaning and method of filling out the dietary habits section of the questionnaire to the patients. However, this study has some strengths. To account for potential variations in dietary habits, participants were recruited from five hospitals located throughout Croatia. Furthermore, 36 dietary variables were derived from the 141-item FFQ for the calculation of the DII^®^ score, which allowed the inclusion of a broader range of dietary patterns and variables.

## 5. Conclusions

In the current study, lower TSH levels, higher free T4 levels, and lower BMI values were observed in subjects with HT and a more anti-inflammatory diet. Moreover, a significant positive association between DII^®^ and TSH was detected, which was maintained after adjustment for age, gender, caloric consumption, and physical activity. An anti-inflammatory diet appears to have beneficial effects in individuals with Hashimoto’s thyroiditis, indicating that dietary modifications focused on reducing DII^®^ levels might be a viable strategy for improving clinical outcomes in these patients. Our findings imply that dietary inflammatory potential could influence thyroid function and systemic inflammation, providing insights into lifestyle factors affecting inflammation-related conditions. Understanding these associations may help develop targeted dietary strategies to manage thyroid health and reduce inflammation risk. However, further research is needed to validate and verify the causal relationship between DII^®^ and thyroid function.

## Figures and Tables

**Table 1:** Participants’ characteristics by Dietary Inflammatory Index tertiles (mean values and standard deviations; numbers and percentages).

Variables	Dietary Inflammatory Index Tertiles						*p* *
	T1 < −1.4 (*n* = 50)		T2 −1.39 to +1.20 (*n* = 51)		T3 > +1.21 (*n* = 48)		
	Mean	SD	Mean	SD	Mean	SD	
Age (years)	47.52	11.73	48.90	12.68	45.43	14.24	0.42
Body mass index (kg/m^2^)	27.46	4.45	28.74	5.92	28.80	7.46	0.46
	*n*	%	*n*	%	*n*	%	
Gender							
Female	44	88	49	96	47	98	0.09
Marital status							
Unmarried or without a partner	6	12	7	14	7	14.6	0.36
Married or with a partner	38	76	42	82	36	75	
Divorced	6	12	2	4	3	6.3	
Widowed	0	0	0	0	2	4.1	
Education level							
High school	3	6	8	16	5	10.4	0.54
College	34	68	34	66	33	68.8	
University	13	26	9	18	10	20.8	
Occupation level							
Employed	35	70	32	62	35	72.9	0.87
Unemployed	4	8	8	16	5	10.4	
Student	2	4	2	4	1	2.1	
Retired	9	18	9	18	7	14.6	
Smoking							0.82
Yes	15	30	15	30	13	27	
No	35	70	36	70	35	73	
Use of anti-inflammatory medications							0.17
Yes	17	34	27	52	23	47.9	
No	33	66	24	47	25	52	
Physical activity level							
Low	14	28	29	56	15	31.3	0.008
Moderate	33	66	19	38	33	68.7	
High	3	6	3	6	0	0	

DII^®^ T1, T2, T3 Dietary Inflammatory Index tertiles. * *p* < 0.05.

**Table 2:** Distribution of C-reactive protein and thyroid function markers across categories of Dietary Inflammatory Index tertiles (mean values and standard deviations).

Variables	Dietary Inflammatory Index Tertiles						*p* *
	T1 < −1.4 (*n* = 50)		T2 −1.39 to +1.20 (*n* = 51)		T3 > +1.21 (*n* = 48)		
	Mean	SD	Mean	SD	Mean	SD	
CRP (nmol/L)	3.09	1.74	3.46	4.21	4.09	5.21	0.46
Thyroid-stimulating hormone (mIU/L)	2.56	1.71	3.89	4.99	3.97	3.15	0.09
Free thyroxine (pmol/L)	14.88	2.33	13.05	3.46	13.95	2.65	0.007
Thyroid peroxidase antibodies (IU/L)	232.92	131.65	650.52	1715.31	603.25	528.76	0.09

T1, T2, T3 Dietary Inflammatory Index tertiles; CRP-C-reactive protein. * *p* < 0.05.

**Table 3:** Participants’ energy and nutrient intakes by Dietary Inflammatory Index tertiles (mean values and standard deviations).

Variables	Dietary Inflammatory Index Tertiles						*p*
	T1 < −1.4 (*n* = 50)		T2 −1.39 to +1.20 (*n* = 51)		T3 > +1.21 (*n* = 48)		
	Mean	SD	Mean	SD	Mean	SD	
DII^®^ score	−2.74	0.75	−0.28	0.67	3.16	1.52	<0.001 *
Energy intake—EI (kJ)	11.98	3.17	9.71	3.52	7.00	2.27	<0.001 *
Proteins (% EI)	16.04	1.96	16.77	3.35	18.09	4.84	0.02 *
Total fat (% EI)	37.45	6.45	39.22	7.70	40.10	7.06	0.17
Saturated fat (% EI)	14.27	2.99	16.02	3.80	15.09	3.62	0.04 *
Monounsaturated fatty acids (% EI)	15.31	4.30	15.42	4.86	15.07	2.23	0.91
Polyunsaturated fatty acids (% EI)	7.18	1.67	6.59	2.00	7.53	3.25	0.15
Omega 3 fatty acids (g)	1.43	1.22	0.78	0.79	0.43	0.26	<0.001 *
Omega 6 fatty acids (g)	1.83	1.33	1.75	1.45	1.56	1.33	0.002 *
Trans fatty acids (% EI)	1.74	0.68	1.71	1.11	1.21	0.64	0.003 *
Cholesterol (mg)	379.15	124.94	323.36	162.54	259.10	126.67	0.002 *
Carbohydrates (% EI)	43.65	6.98	43.05	9.53	39.29	8.36	<0.001 *
Dietary fiber (g)	43.73	13.25	37.94	20.76	15.92	6.00	<0.001 *
Alcohol (g)	2.86	3.88	0.97	2.26	2.51	7.86	0.16
Beta-carotene (mg)	5.13	3.90	3.20	3.32	3.04	2.17	<0.001 *
Vitamin A (mg)	1.34	1.0	1.1	0.8	0.59	0.52	<0.001 *
Vitamin D (µg)	10.55	12.39	8.08	10.77	4.28	5.23	0.01 *
Vitamin E (mg)	10.71	8.31	7.91	7.36	6.91	4.23	<0.001 *
Vitamin C (mg)	216.03	129.35	152.37	129.00	106.34	53.29	<0.001*
Vitamin B_1_ (mg)	0.84	3.55	0.85	2.88	0.61	1.57	<0.001*
Vitamin B_2_ (mg)	2.15	1.11	1.88	1.06	1.07	0.62	<0.001 *
Vitamin B_3_ (mg)	26.49	7.28	23.39	9.24	14.97	6.26	<0.001 *
Vitamin B_6_ (mg)	4.57	2.25	3.02	1.88	1.96	1.03	<0.001 *
Vitamin B_9_ (mg)	477.99	175.33	369.55	171.31	203.66	80.19	<0.001 *
Vitamin B_12_ (µg)	6.73	3.11	5.13	3.33	3.59	2.31	<0.001 *
Calcium (mg)	1501.60	506.55	1235.39	571.85	684.38	343.82	<0.001 *
Iron (mg)	19.87	8.35	15.41	10.30	8.93	3.64	<0.001 *
Magnesium (mg)	539.10	196.24	386.75	139.95	218.11	75.44	<0.001 *
Zinc (mg)	17.54	4.24	14.94	6.06	10.01	4.26	<0.001 *
Selenium (µg)	38.54	15.26	28.11	17.66	21.36	11.27	<0.001 *
Iodine (µg)	77.40	37.51	65.76	41.62	37.86	25.59	<0.001 *
Caffeine (mg)	302.01	260.08	284.12	226.35	273.58	248.98	0.85
Flavan-3-ol (mg)	835.08	1158.95	235.84	523.42	466.40	1581.19	0.03 *
Flavones (mg)	4.02	2.17	3.19	2.38	1.48	1.18	<0.001 *
Flavonols (mg)	104.92	92.79	74.00	54.13	43.43	51.40	<0.001 *
Flavonones (mg)	85.23	94.60	60.95	82.26	27.86	38.49	0.001 *
Anthocyanidins (mg)	57.46	49.14	21.23	29.32	21.65	56.22	<0.001 *

T1, T2, T3 Dietary Inflammatory Index tertiles. * *p* < 0.05.

**Table 4:** Standardized regression coefficients (β) of the association between DII^®^ score and anthropometric parameters, C-reactive protein, and thyroid function markers.

Variables	Model 0 ^†^		Model I ^‡^	
	β	*p*	β	*p*
Body weight (kg)	−0.01	0.70	0.01	0.79
Body mass index (kg/m^2^)	0.06	0.46	0.14	0.04 *
C-reactive protein (nmol/L)	0.20	0.24	0.03	0.52
Thyroid-stimulating hormone (mIU/L)	0.42	<0.001 *	0.33	0.002 *
Free thyroxine (pmol/L)	0.19	<0.001 *	0.02	0.68
Thyroid peroxidase antibodies (IU/L)	0.04	0.52	−0.02	0.69

* Statistical significance at *p* < 0.05. ^†^ Model 0, linear regression analysis without adjustment. ^‡^ Model I, linear regression analysis with adjustment for age, gender, energy intake, and physical activity.

## Data Availability

The original contributions presented in the study are included in the article, further inquiries can be directed to the corresponding author.
